# 4,5,7,8,17-Penta­hydr­oxy-14,18-dimethyl-6-methyl­ene-3,10-dioxapenta­cyclo­[9.8.0.0^1,7^.0^4,19^.0^13,18^]nona­dec-14-ene-9,16-dione methanol solvate dihydrate

**DOI:** 10.1107/S1600536809010502

**Published:** 2009-03-28

**Authors:** Chin Hoe Teh, Siew Chin Teoh, Chin Sing Yeap, Kit Lam Chan, Hoong-Kun Fun

**Affiliations:** a School of Pharmaceutical Sciences, Universiti Sains Malaysia, 11800 USM, Penang, Malaysia; b X-ray Crystallography Unit, School of Physics, Universiti Sains Malaysia, 11800 USM, Penang, Malaysia

## Abstract

The title quassinoid compound, C_20_H_24_O_9_·CH_3_OH·2H_2_O, is a natural eurycomanone isolated from the roots of *Eurycoma longifolia*. The mol­ecules contain a fused five-ring system, with one tetra­hydro­furan ring adopting an envelope conformation, one tetra­hydro­pyran-2-one ring in a screw boat conformation, one cyclo­hexenone ring in a half-chair conformation and two cyclo­hexane rings in chair conformations. Intra­molecular C—H⋯O inter­actions generate *S*(5) ring motifs and an O—H⋯O inter­action generates an *S*(7) ring motif. In the crystal, mol­ecules are linked *via* inter­molecular O—H⋯O inter­actions along the *b* axis and further stacked along *a* axis. The absolute configuration of the title compound was inferred from previously solved structures of its analogues.

## Related literature

For bond-length data, see Allen *et al.* (1987 ▶). For hydrogen-bond motifs, see Bernstein *et al.* (1995 ▶). For ring conformations, see Cremer & Pople (1975 ▶). For quassinoids and bioactivity, see Itokawa *et al.* (1993 ▶); Chan *et al.* (1992 ▶); Kardono *et al.* (1991 ▶); Itokawa *et al.* (1992 ▶); Morita *et al.* (1992 ▶); Morita *et al.* (1993 ▶); Tada *et al.* (1991 ▶); Ang *et al.* (1995 ▶); Chan *et al.* (2004 ▶). For the stability of the temperature controller used for the data collection, see: Cosier & Glazer (1986 ▶).
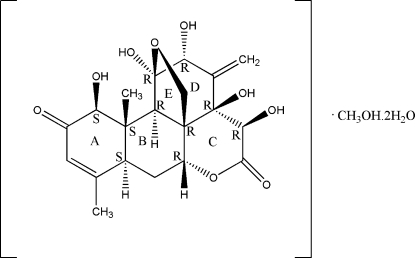


## Experimental

### 

#### Crystal data

C_20_H_24_O_9_·CH_4_O·2H_2_O
*M* *_r_* = 476.47
Orthorhombic, 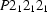

*a* = 9.1817 (1) Å
*b* = 10.7806 (2) Å
*c* = 21.7817 (3) Å
*V* = 2156.04 (6) Å^3^
*Z* = 4
Mo *K*α radiation
μ = 0.12 mm^−1^
*T* = 100 K
0.43 × 0.28 × 0.11 mm

#### Data collection

Bruker SMART APEXII CCD area-detector diffractometer
Absorption correction: multi-scan (**SADABS**; Bruker, 2005 ▶) *T*_min_ = 0.950, *T*_max_ = 0.987
27636 measured reflections
3577 independent reflections
3352 reflections with *I* > 2σ(*I*)
*R*_int_ = 0.033

#### Refinement

*R*[*F* ^2^ > 2σ(*F* ^2^)] = 0.043
*wR*(*F* ^2^) = 0.127
*S* = 1.09
3577 reflections
307 parameters
H-atom parameters constrained
Δρ_max_ = 1.08 e Å^−3^
Δρ_min_ = −0.46 e Å^−3^

### 

Data collection: *APEX2* (Bruker, 2005 ▶); cell refinement: *SAINT* (Bruker, 2005 ▶); data reduction: *SAINT*; program(s) used to solve structure: *SHELXTL* (Sheldrick, 2008 ▶); program(s) used to refine structure: *SHELXTL*; molecular graphics: *SHELXTL*; software used to prepare material for publication: *SHELXTL* and *PLATON* (Spek, 2009 ▶).

## Supplementary Material

Crystal structure: contains datablocks global, I. DOI: 10.1107/S1600536809010502/at2747sup1.cif

Structure factors: contains datablocks I. DOI: 10.1107/S1600536809010502/at2747Isup2.hkl

Additional supplementary materials:  crystallographic information; 3D view; checkCIF report

## Figures and Tables

**Table 1:** Hydrogen-bond geometry (Å, °)

*D*—H⋯*A*	*D*—H	H⋯*A*	*D*⋯*A*	*D*—H⋯*A*
O1*W*—H1*W*1⋯O4^i^	0.88	1.94	2.810 (2)	169
O2—H2⋯O2*W*^ii^	0.82	1.85	2.656 (3)	169
O1*W*—H2*W*1⋯O3^iii^	0.84	2.06	2.873 (3)	163
O3—H3⋯O2	0.82	1.71	2.525 (2)	171
O2*W*—H1*W*2⋯O8	0.95	2.03	2.950 (3)	164
O2*W*—H2*W*2⋯O10	0.85	1.91	2.760 (3)	179
O5—H5⋯O3^iv^	0.82	1.99	2.805 (2)	172
O6—H6⋯O9^v^	0.82	2.14	2.848 (2)	144
O7—H7⋯O1*W*^vi^	0.82	1.84	2.653 (3)	171
O10—H10⋯O7^vii^	0.82	2.29	3.011 (3)	147
O10—H10⋯O8^vii^	0.82	2.23	2.911 (3)	140
C1—H1*A*⋯O9	0.98	2.48	2.936 (3)	108
C1—H1*A*⋯O1^iii^	0.98	2.56	3.507 (3)	162
C7—H7*A*⋯O5	0.98	2.38	2.856 (3)	109
C12—H12*A*⋯O1^iii^	0.98	2.47	3.168 (3)	128
C17—H17*A*⋯O10^v^	0.97	2.60	3.428 (3)	144
C17—H17*B*⋯O6	0.97	2.50	2.929 (3)	107
C19—H19*B*⋯O2	0.96	2.56	2.957 (3)	105

Symmetry codes: (i) 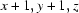; (ii) 
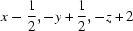
; (iii) 
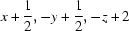
; (iv) 
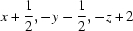
; (v) 
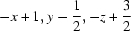
; (vi) 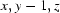; (vii) 
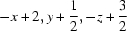
.

**Fig. 1.**
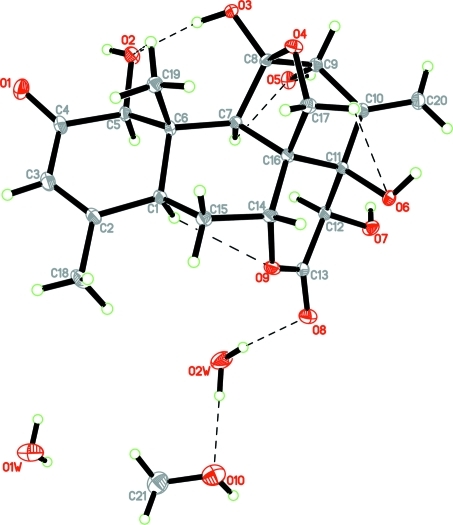
The molecular structure of the title compound with atom labels and 50% probability ellipsoids for non-H atoms. Intramolecular hydrogen bonds are shown as dashed lines.

**Fig. 2.**
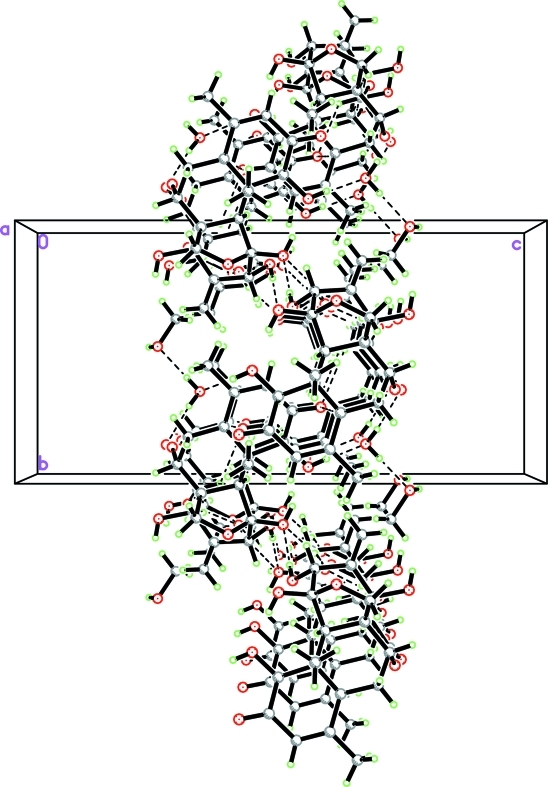
The crystal packing of (I), shows that the molecules were linked via intermolecular O—H···O interactions along b axis and further stacked along a axis. Intermolecular interactions are drawn as dashed lines.

## supplementary crystallographic information

### Comment

*Eurycoma longifolia* Jack is a tall, slender shrub-tree, commonly found in lowland forests below 500 meters above sea level in Southeast Asia.  The roots of this Simaroubaceae plant are used in folk medicine for intermittent fever (malaria), dysentery, glandular swelling and aphrodisiac properties. Various classes of chemical constituents (Itokawa *et al.*, 1993, Chan *et al.*, 1992, Kardono *et al.*, 1991, Itokawa *et al.*, 1992, Morita *et al.*, 1992, Morita *et al.*, 1993) have been identified and some have shown antiulcer (Tada *et al.*, 1991), cytotoxic (Kardono *et al.*, 1991, Itokawa *et al.*, 1992) and antimalarial (Ang *et al.*, 1995, Chan *et al.*, 2004) activities. In our continuing search for the bioactive compounds from *E. longifolia*, we have isolated eurycomanone (1), a quassinoid in crystalline form.

The title compound (Fig. 1), contains quassinoid, one molecule of methanol and two molecules of water solvents. The bond lengths and angles are within normal ranges (Allen *et al.*, 1987). The molecule of the title compound contains a fused five-ring system A/B/C/D/E (Scheme 1). The A/B and B/D junctions are trans-fused, whereas B/C, B/E and C/D are cis-fused (Fig 1). The cyclohexenone ring A (C1—C6) has a half-chair conformation with puckering parameters of Q = 0.507 (2) Å, Θ = 130.2 (3)° and φ = 97.3 (4)°. The cyclohexane ring B (C1-C6-C7-C16-C14-C15) and D (C7-C8-C9-C10-C11-C16) adopt a chair conformation with puckering parameters of Q = 0.565 (2) Å, Θ = 154.6 (3)° and φ = 179.8 (6)° and Q = 0.660 (2) Å, Θ = 151.68 (17)° and φ = 183.1 (4)° respectively. The tetrahydro-pyran-2-one ring C (O9-C13-C12-C11-C16-14) has a screw boat conformation with puckering parameters Q = 0.502 (3) Å, Θ = 38.4 (2)° and φ = 193.4 (5)°. The tetrahydro-furan ring E (C7-C8-O4-C17-C16) is in an envelope conformation with puckering parameters of Q = 0.450 (2) Å and φ = 254.5 (3)°.

The intramolecular interactions C1—H1A···O9, C17—H17B···O6, C19—H19B···O2 and C7—H7A···O5 generate S(5) ring motifs, and O3—H3···O2 generates an S(7) ring motif (Bernstein *et al.*, 1995). The crystal packing shows that the molecules were linked via intermolecular O—H···O interactions along *b* axis and further stacked along *a* axis (Fig 2). The absolute configuration of the title compound was inferred from previously solved structures of its analogues (Tada *et al.*, 1991).

### Experimental

The air-dried powdered roots of *E. longifolia* (11.6 kg) were extracted with MeOH. The MeOH extract on evaporation to dryness yielded 485 g of dark brown residue which was next chromatographed on a Diaion HP 20 column using H_2_O-MeOH (1:0 - 0:1) gradient mixtures to afford 4 fractions (Fr 1 - 4). Fr 2 was concentrated under vacuum to give 52.2 g of residue. The residue was resuspended in water and then partitioned successively with ethyl acetate and saturated *n*-butanol to yield three subfractions. The *n*-BuOH subfraction (20.4 g) was further fractionated on a silica gel column using CHCl_3_-MeOH (1:0 - 1:1) gradient mixtures to obtain 7 portions (A1-A7). A3 was further purified by centrifugal silica gel TLC with CHCl_3_-MeOH (1:0 - 1:1) gradient mixtures. Upon solvent removal, the residue obtained on subsequent recrystallization from CHCl_3_-MeOH (9:1, v/v) at room temperature afforded 1 as colourless crystals (75.3 mg).

### Refinement

The H atoms bound to O1W and O2W were located from the difference Fourier map and constrained to ride with the parent atom with U_iso_(H)= 1.5 U_eq_(O). The H atoms of the hydroxy groups were positioned by a freely rotating O—H bond and constrained with a fixed distance of 0.82 Å. The rest of the hydrogen atoms were positioned geometrically with a riding model approximation with C—H = 0.93-0.98 Å and U_iso_(H) = 1.2 or 1.5 U_eq_(C). A rotating-group model was used for the hydrogen of the methyl groups. As there are not enough anomalous dispersion to determine the aboslute configuration, 2770 Friedel pairs were merged before final refinement. The absolute stereochemistry of eurycomanone was inferred following those reported (Tada *et al.*, 1991) for its analogues.

### Crystal data

**Table:**

C_20_H_24_O_9_·CH_4_O·2H_2_O	*F*(000) = 1016
*M**_r_* = 476.47	*D*_x_ = 1.468 Mg m^−^^3^
Orthorhombic, *P*2_1_2_1_2_1_	Mo *K*α radiation, λ = 0.71073 Å
Hall symbol: P 2ac 2ab	Cell parameters from 9889 reflections
*a* = 9.1817 (1) Å	θ = 2.4–30.1°
*b* = 10.7806 (2) Å	µ = 0.12 mm^−^^1^
*c* = 21.7817 (3) Å	*T* = 100 K
*V* = 2156.04 (6) Å^3^	Block, colourless
*Z* = 4	0.43 × 0.28 × 0.11 mm

### Data collection

**Table:**

Bruker SMART APEXII CCD area-detector diffractometer	3577 independent reflections
Radiation source: fine-focus sealed tube	3352 reflections with *I* > 2σ(*I*)
graphite	*R*_int_ = 0.033
φ and ω scans	θ_max_ = 30.1°, θ_min_ = 1.9°
Absorption correction: multi-scan (*SADABS*; Bruker, 2005)	*h* = −12→12
*T*_min_ = 0.950, *T*_max_ = 0.987	*k* = −15→15
27636 measured reflections	*l* = −29→30

### Refinement

**Table:**

Refinement on *F*^2^	Primary atom site location: structure-invariant direct methods
Least-squares matrix: full	Secondary atom site location: difference Fourier map
*R*[*F*^2^ > 2σ(*F*^2^)] = 0.043	Hydrogen site location: inferred from neighbouring sites
*wR*(*F*^2^) = 0.127	H-atom parameters constrained
*S* = 1.09	*w* = 1/[σ^2^(*F*_o_^2^) + (0.0739*P*)^2^ + 1.2123*P*] where *P* = (*F*_o_^2^ + 2*F*_c_^2^)/3
3577 reflections	(Δ/σ)_max_ < 0.001
307 parameters	Δρ_max_ = 1.08 e Å^−^^3^
0 restraints	Δρ_min_ = −0.46 e Å^−^^3^

### Special details

**Table:**

Experimental. The crystal was placed in the cold stream of an Oxford Cyrosystems Cobra open-flow nitrogen cryostat (Cosier & Glazer, 1986) operating at 100.0 (1)K.
Geometry. All esds (except the esd in the dihedral angle between two l.s. planes) are estimated using the full covariance matrix. The cell esds are taken into account individually in the estimation of esds in distances, angles and torsion angles; correlations between esds in cell parameters are only used when they are defined by crystal symmetry. An approximate (isotropic) treatment of cell esds is used for estimating esds involving l.s. planes.
Refinement. Refinement of F^2^ against ALL reflections. The weighted R-factor wR and goodness of fit S are based on F^2^, conventional R-factors R are based on F, with F set to zero for negative F^2^. The threshold expression of F^2^ > 2sigma(F^2^) is used only for calculating R-factors(gt) etc. and is not relevant to the choice of reflections for refinement. R-factors based on F^2^ are statistically about twice as large as those based on F, and R- factors based on ALL data will be even larger.

### Fractional atomic coordinates and isotropic or equivalent isotropic displacement parameters (Å^2^)

**Table:**

	*x*	*y*	*z*	*U*_iso_*/*U*_eq_	
O1	0.1926 (2)	0.29462 (17)	1.07263 (8)	0.0182 (4)	
O2	0.3034 (2)	0.07030 (16)	1.05042 (8)	0.0175 (4)	
H2	0.3223	0.0997	1.0842	0.026*	
O3	0.28241 (19)	−0.14352 (15)	1.00393 (7)	0.0117 (3)	
H3	0.2817	−0.0757	1.0210	0.018*	
O4	0.22993 (18)	−0.17614 (15)	0.90312 (7)	0.0118 (3)	
O5	0.58130 (19)	−0.16373 (15)	0.97902 (7)	0.0129 (3)	
H5	0.6465	−0.2146	0.9843	0.019*	
O6	0.5152 (2)	−0.13426 (15)	0.77017 (7)	0.0127 (3)	
H6	0.4834	−0.2045	0.7648	0.019*	
O7	0.78562 (19)	−0.09069 (17)	0.82090 (8)	0.0147 (3)	
H7	0.8241	−0.1258	0.8501	0.022*	
O8	0.74680 (19)	0.14137 (17)	0.77962 (8)	0.0167 (4)	
O9	0.51228 (19)	0.13846 (15)	0.79549 (7)	0.0126 (3)	
C1	0.3189 (3)	0.22030 (19)	0.89489 (10)	0.0101 (4)	
H1A	0.4255	0.2249	0.8946	0.012*	
C2	0.2671 (3)	0.3479 (2)	0.91422 (11)	0.0130 (4)	
C3	0.2330 (3)	0.3740 (2)	0.97285 (11)	0.0152 (4)	
H3A	0.2023	0.4536	0.9830	0.018*	
C4	0.2431 (3)	0.2802 (2)	1.02085 (11)	0.0133 (4)	
C5	0.3254 (3)	0.1614 (2)	1.00468 (10)	0.0112 (4)	
H5A	0.4296	0.1810	1.0035	0.013*	
C6	0.2803 (2)	0.11364 (19)	0.94038 (10)	0.0093 (4)	
C7	0.3786 (3)	0.00071 (19)	0.92185 (9)	0.0086 (4)	
H7A	0.4783	0.0189	0.9351	0.010*	
C8	0.3392 (2)	−0.1308 (2)	0.94491 (9)	0.0094 (4)	
C9	0.4726 (3)	−0.21530 (19)	0.93946 (9)	0.0106 (4)	
H9A	0.4470	−0.2984	0.9542	0.013*	
C10	0.5283 (3)	−0.2248 (2)	0.87386 (10)	0.0108 (4)	
C11	0.5208 (3)	−0.10845 (19)	0.83436 (9)	0.0094 (4)	
C12	0.6596 (3)	−0.0292 (2)	0.84086 (10)	0.0105 (4)	
H12A	0.6718	−0.0059	0.8840	0.013*	
C13	0.6438 (3)	0.0881 (2)	0.80244 (10)	0.0118 (4)	
C14	0.3716 (3)	0.0779 (2)	0.80774 (10)	0.0104 (4)	
H14A	0.3327	0.0472	0.7687	0.012*	
C15	0.2754 (3)	0.1834 (2)	0.82945 (10)	0.0119 (4)	
H15A	0.1742	0.1576	0.8288	0.014*	
H15B	0.2861	0.2540	0.8022	0.014*	
C16	0.3828 (3)	−0.0312 (2)	0.85175 (10)	0.0094 (4)	
C17	0.2494 (3)	−0.1166 (2)	0.84404 (10)	0.0118 (4)	
H17A	0.1638	−0.0687	0.8332	0.014*	
H17B	0.2669	−0.1779	0.8123	0.014*	
C18	0.2638 (3)	0.4474 (2)	0.86586 (12)	0.0194 (5)	
H18A	0.2506	0.5268	0.8850	0.029*	
H18B	0.1846	0.4319	0.8381	0.029*	
H18C	0.3540	0.4469	0.8436	0.029*	
C19	0.1144 (3)	0.0868 (2)	0.94083 (11)	0.0125 (4)	
H19A	0.0618	0.1637	0.9423	0.019*	
H19B	0.0904	0.0377	0.9762	0.019*	
H19C	0.0883	0.0423	0.9043	0.019*	
C20	0.5852 (3)	−0.3310 (2)	0.85366 (11)	0.0154 (5)	
H20A	0.5908	−0.3992	0.8797	0.018*	
H20B	0.6192	−0.3368	0.8136	0.018*	
O10	0.9816 (2)	0.5246 (2)	0.75796 (10)	0.0265 (4)	
H10	1.0588	0.5227	0.7390	0.040*	
C21	0.9754 (3)	0.6332 (3)	0.79372 (14)	0.0273 (6)	
H21A	0.8955	0.6276	0.8220	0.041*	
H21B	0.9619	0.7037	0.7674	0.041*	
H21C	1.0648	0.6424	0.8162	0.041*	
O1W	0.9259 (2)	0.8195 (2)	0.91766 (10)	0.0283 (5)	
H1W1	1.0216	0.8137	0.9173	0.042*	
H2W1	0.8959	0.7574	0.9371	0.042*	
O2W	0.8990 (3)	0.3466 (2)	0.84211 (9)	0.0341 (6)	
H1W2	0.8346	0.2908	0.8221	0.051*	
H2W2	0.9261	0.4012	0.8161	0.051*	

### Atomic displacement parameters (Å^2^)

**Table:**

	*U*^11^	*U*^22^	*U*^33^	*U*^12^	*U*^13^	*U*^23^
O1	0.0210 (9)	0.0187 (8)	0.0150 (8)	0.0028 (7)	0.0020 (7)	−0.0043 (7)
O2	0.0321 (10)	0.0119 (7)	0.0086 (7)	0.0001 (7)	0.0005 (7)	−0.0005 (6)
O3	0.0158 (8)	0.0101 (7)	0.0092 (7)	0.0001 (6)	0.0037 (6)	0.0008 (5)
O4	0.0128 (7)	0.0118 (7)	0.0108 (7)	−0.0035 (6)	−0.0018 (6)	0.0019 (6)
O5	0.0141 (8)	0.0127 (7)	0.0121 (7)	0.0033 (6)	−0.0032 (6)	−0.0012 (6)
O6	0.0206 (8)	0.0112 (7)	0.0063 (6)	−0.0015 (7)	0.0009 (6)	−0.0017 (5)
O7	0.0134 (8)	0.0162 (8)	0.0146 (7)	0.0044 (7)	0.0038 (6)	0.0033 (6)
O8	0.0171 (9)	0.0142 (7)	0.0187 (8)	−0.0025 (7)	0.0036 (7)	0.0023 (6)
O9	0.0142 (8)	0.0098 (7)	0.0136 (7)	−0.0001 (6)	0.0011 (6)	0.0032 (6)
C1	0.0122 (9)	0.0079 (8)	0.0102 (9)	0.0010 (7)	0.0000 (8)	0.0003 (7)
C2	0.0134 (10)	0.0084 (9)	0.0173 (10)	0.0004 (8)	−0.0002 (8)	0.0008 (8)
C3	0.0159 (11)	0.0111 (9)	0.0186 (11)	0.0014 (8)	0.0004 (9)	−0.0026 (8)
C4	0.0139 (11)	0.0100 (9)	0.0159 (10)	0.0005 (8)	−0.0012 (8)	−0.0036 (8)
C5	0.0152 (10)	0.0090 (9)	0.0093 (9)	0.0017 (8)	−0.0001 (8)	−0.0021 (7)
C6	0.0113 (9)	0.0073 (8)	0.0093 (8)	0.0015 (8)	−0.0011 (7)	−0.0009 (7)
C7	0.0107 (9)	0.0080 (8)	0.0071 (8)	0.0001 (7)	−0.0005 (8)	−0.0001 (7)
C8	0.0102 (9)	0.0105 (9)	0.0075 (8)	−0.0004 (8)	0.0010 (7)	−0.0001 (7)
C9	0.0141 (10)	0.0088 (8)	0.0088 (8)	0.0002 (8)	0.0002 (8)	0.0002 (7)
C10	0.0142 (10)	0.0093 (8)	0.0089 (9)	0.0000 (8)	0.0001 (8)	−0.0006 (7)
C11	0.0135 (10)	0.0085 (8)	0.0063 (8)	0.0000 (8)	−0.0001 (8)	−0.0007 (7)
C12	0.0134 (10)	0.0094 (9)	0.0088 (8)	0.0001 (8)	0.0000 (8)	−0.0001 (7)
C13	0.0158 (11)	0.0100 (9)	0.0097 (9)	−0.0014 (8)	0.0013 (8)	−0.0020 (7)
C14	0.0128 (10)	0.0103 (9)	0.0081 (9)	−0.0009 (8)	−0.0012 (8)	0.0008 (7)
C15	0.0151 (10)	0.0112 (9)	0.0094 (9)	−0.0001 (8)	−0.0015 (8)	0.0013 (7)
C16	0.0130 (10)	0.0080 (9)	0.0072 (8)	−0.0012 (8)	−0.0015 (7)	0.0003 (7)
C17	0.0132 (10)	0.0128 (10)	0.0094 (9)	−0.0018 (8)	−0.0015 (8)	0.0003 (7)
C18	0.0282 (13)	0.0100 (9)	0.0199 (11)	0.0022 (9)	−0.0016 (10)	0.0030 (8)
C19	0.0107 (10)	0.0120 (9)	0.0147 (10)	0.0004 (8)	0.0005 (8)	−0.0008 (8)
C20	0.0230 (12)	0.0115 (10)	0.0116 (9)	0.0034 (9)	0.0007 (9)	−0.0010 (8)
O10	0.0200 (10)	0.0323 (11)	0.0272 (10)	−0.0057 (9)	−0.0002 (8)	0.0013 (8)
C21	0.0211 (13)	0.0325 (14)	0.0283 (13)	0.0028 (12)	0.0014 (11)	−0.0004 (12)
O1W	0.0188 (9)	0.0352 (11)	0.0310 (10)	−0.0017 (9)	−0.0018 (8)	0.0140 (9)
O2W	0.0531 (15)	0.0298 (11)	0.0195 (9)	−0.0224 (11)	0.0129 (10)	−0.0082 (8)

### Geometric parameters (Å, °)

**Table:**

O1—C4	1.229 (3)	C9—C10	1.521 (3)
O2—C5	1.414 (3)	C9—H9A	0.9800
O2—H2	0.8200	C10—C20	1.333 (3)
O3—C8	1.394 (3)	C10—C11	1.523 (3)
O3—H3	0.8200	C11—C12	1.541 (3)
O4—C8	1.440 (3)	C11—C16	1.562 (3)
O4—C17	1.449 (3)	C12—C13	1.523 (3)
O5—C9	1.431 (3)	C12—H12A	0.9800
O5—H5	0.8200	C14—C15	1.516 (3)
O6—C11	1.426 (2)	C14—C16	1.520 (3)
O6—H6	0.8200	C14—H14A	0.9800
O7—C12	1.402 (3)	C15—H15A	0.9700
O7—H7	0.8200	C15—H15B	0.9700
O8—C13	1.213 (3)	C16—C17	1.542 (3)
O9—C13	1.333 (3)	C17—H17A	0.9700
O9—C14	1.472 (3)	C17—H17B	0.9700
C1—C2	1.516 (3)	C18—H18A	0.9600
C1—C15	1.533 (3)	C18—H18B	0.9600
C1—C6	1.559 (3)	C18—H18C	0.9600
C1—H1A	0.9800	C19—H19A	0.9600
C2—C3	1.345 (3)	C19—H19B	0.9600
C2—C18	1.503 (3)	C19—H19C	0.9600
C3—C4	1.458 (3)	C20—H20A	0.9300
C3—H3A	0.9300	C20—H20B	0.9300
C4—C5	1.528 (3)	O10—C21	1.408 (4)
C5—C6	1.549 (3)	O10—H10	0.8200
C5—H5A	0.9800	C21—H21A	0.9600
C6—C19	1.551 (3)	C21—H21B	0.9600
C6—C7	1.568 (3)	C21—H21C	0.9600
C7—C8	1.547 (3)	O1W—H1W1	0.8814
C7—C16	1.566 (3)	O1W—H2W1	0.8396
C7—H7A	0.9800	O2W—H1W2	0.9487
C8—C9	1.531 (3)	O2W—H2W2	0.8533
			
C5—O2—H2	109.5	C10—C11—C16	109.83 (18)
C8—O3—H3	109.5	C12—C11—C16	110.67 (17)
C8—O4—C17	108.97 (16)	O7—C12—C13	107.48 (18)
C9—O5—H5	109.5	O7—C12—C11	113.11 (18)
C11—O6—H6	109.5	C13—C12—C11	109.31 (18)
C12—O7—H7	109.5	O7—C12—H12A	109.0
C13—O9—C14	126.43 (17)	C13—C12—H12A	109.0
C2—C1—C15	114.35 (19)	C11—C12—H12A	109.0
C2—C1—C6	114.97 (18)	O8—C13—O9	117.8 (2)
C15—C1—C6	109.89 (17)	O8—C13—C12	123.0 (2)
C2—C1—H1A	105.6	O9—C13—C12	119.14 (19)
C15—C1—H1A	105.6	O9—C14—C15	103.60 (17)
C6—C1—H1A	105.6	O9—C14—C16	113.43 (18)
C3—C2—C18	120.8 (2)	C15—C14—C16	115.04 (18)
C3—C2—C1	121.8 (2)	O9—C14—H14A	108.2
C18—C2—C1	117.3 (2)	C15—C14—H14A	108.2
C2—C3—C4	121.4 (2)	C16—C14—H14A	108.2
C2—C3—H3A	119.3	C14—C15—C1	109.45 (18)
C4—C3—H3A	119.3	C14—C15—H15A	109.8
O1—C4—C3	123.1 (2)	C1—C15—H15A	109.8
O1—C4—C5	120.3 (2)	C14—C15—H15B	109.8
C3—C4—C5	116.6 (2)	C1—C15—H15B	109.8
O2—C5—C4	110.43 (18)	H15A—C15—H15B	108.2
O2—C5—C6	111.59 (18)	C14—C16—C17	109.82 (18)
C4—C5—C6	110.77 (18)	C14—C16—C11	108.34 (18)
O2—C5—H5A	108.0	C17—C16—C11	107.41 (17)
C4—C5—H5A	108.0	C14—C16—C7	116.32 (17)
C6—C5—H5A	108.0	C17—C16—C7	102.56 (17)
C5—C6—C19	108.61 (18)	C11—C16—C7	111.96 (17)
C5—C6—C1	105.59 (16)	O4—C17—C16	105.42 (17)
C19—C6—C1	111.42 (18)	O4—C17—H17A	110.7
C5—C6—C7	109.69 (17)	C16—C17—H17A	110.7
C19—C6—C7	114.94 (18)	O4—C17—H17B	110.7
C1—C6—C7	106.17 (17)	C16—C17—H17B	110.7
C8—C7—C16	96.96 (16)	H17A—C17—H17B	108.8
C8—C7—C6	119.59 (18)	C2—C18—H18A	109.5
C16—C7—C6	115.84 (17)	C2—C18—H18B	109.5
C8—C7—H7A	107.9	H18A—C18—H18B	109.5
C16—C7—H7A	107.9	C2—C18—H18C	109.5
C6—C7—H7A	107.9	H18A—C18—H18C	109.5
O3—C8—O4	106.79 (17)	H18B—C18—H18C	109.5
O3—C8—C9	108.20 (17)	C6—C19—H19A	109.5
O4—C8—C9	107.87 (17)	C6—C19—H19B	109.5
O3—C8—C7	118.47 (17)	H19A—C19—H19B	109.5
O4—C8—C7	105.57 (17)	C6—C19—H19C	109.5
C9—C8—C7	109.47 (18)	H19A—C19—H19C	109.5
O5—C9—C10	110.95 (19)	H19B—C19—H19C	109.5
O5—C9—C8	106.27 (17)	C10—C20—H20A	120.0
C10—C9—C8	112.45 (17)	C10—C20—H20B	120.0
O5—C9—H9A	109.0	H20A—C20—H20B	120.0
C10—C9—H9A	109.0	C21—O10—H10	109.5
C8—C9—H9A	109.0	O10—C21—H21A	109.5
C20—C10—C9	120.0 (2)	O10—C21—H21B	109.5
C20—C10—C11	122.6 (2)	H21A—C21—H21B	109.5
C9—C10—C11	117.39 (18)	O10—C21—H21C	109.5
O6—C11—C10	113.25 (17)	H21A—C21—H21C	109.5
O6—C11—C12	103.16 (17)	H21B—C21—H21C	109.5
C10—C11—C12	111.51 (18)	H1W1—O1W—H2W1	105.9
O6—C11—C16	108.23 (17)	H1W2—O2W—H2W2	108.4
			
C15—C1—C2—C3	148.4 (2)	C20—C10—C11—O6	27.0 (3)
C6—C1—C2—C3	19.9 (3)	C9—C10—C11—O6	−155.1 (2)
C15—C1—C2—C18	−35.2 (3)	C20—C10—C11—C12	−88.9 (3)
C6—C1—C2—C18	−163.7 (2)	C9—C10—C11—C12	89.0 (2)
C18—C2—C3—C4	−176.8 (2)	C20—C10—C11—C16	148.1 (2)
C1—C2—C3—C4	−0.5 (4)	C9—C10—C11—C16	−34.0 (3)
C2—C3—C4—O1	−168.7 (2)	O6—C11—C12—O7	−59.4 (2)
C2—C3—C4—C5	13.3 (3)	C10—C11—C12—O7	62.5 (2)
O1—C4—C5—O2	13.0 (3)	C16—C11—C12—O7	−174.96 (17)
C3—C4—C5—O2	−168.9 (2)	O6—C11—C12—C13	60.3 (2)
O1—C4—C5—C6	137.2 (2)	C10—C11—C12—C13	−177.82 (18)
C3—C4—C5—C6	−44.8 (3)	C16—C11—C12—C13	−55.3 (2)
O2—C5—C6—C19	63.6 (2)	C14—O9—C13—O8	166.4 (2)
C4—C5—C6—C19	−59.9 (2)	C14—O9—C13—C12	−16.6 (3)
O2—C5—C6—C1	−176.83 (19)	O7—C12—C13—O8	−27.5 (3)
C4—C5—C6—C1	59.7 (2)	C11—C12—C13—O8	−150.6 (2)
O2—C5—C6—C7	−62.8 (2)	O7—C12—C13—O9	155.59 (19)
C4—C5—C6—C7	173.71 (18)	C11—C12—C13—O9	32.5 (3)
C2—C1—C6—C5	−48.1 (2)	C13—O9—C14—C15	147.4 (2)
C15—C1—C6—C5	−178.84 (18)	C13—O9—C14—C16	22.0 (3)
C2—C1—C6—C19	69.6 (2)	O9—C14—C15—C1	−73.9 (2)
C15—C1—C6—C19	−61.1 (2)	C16—C14—C15—C1	50.4 (3)
C2—C1—C6—C7	−164.58 (18)	C2—C1—C15—C14	161.25 (19)
C15—C1—C6—C7	64.7 (2)	C6—C1—C15—C14	−67.7 (2)
C5—C6—C7—C8	83.5 (2)	O9—C14—C16—C17	−159.36 (18)
C19—C6—C7—C8	−39.2 (3)	C15—C14—C16—C17	81.6 (2)
C1—C6—C7—C8	−162.86 (18)	O9—C14—C16—C11	−42.3 (2)
C5—C6—C7—C16	−161.04 (18)	C15—C14—C16—C11	−161.36 (18)
C19—C6—C7—C16	76.2 (2)	O9—C14—C16—C7	84.8 (2)
C1—C6—C7—C16	−47.4 (2)	C15—C14—C16—C7	−34.2 (3)
C17—O4—C8—O3	154.14 (17)	O6—C11—C16—C14	−51.0 (2)
C17—O4—C8—C9	−89.8 (2)	C10—C11—C16—C14	−175.06 (18)
C17—O4—C8—C7	27.2 (2)	C12—C11—C16—C14	61.4 (2)
C16—C7—C8—O3	−161.90 (19)	O6—C11—C16—C17	67.6 (2)
C6—C7—C8—O3	−36.9 (3)	C10—C11—C16—C17	−56.5 (2)
C16—C7—C8—O4	−42.4 (2)	C12—C11—C16—C17	179.97 (17)
C6—C7—C8—O4	82.6 (2)	O6—C11—C16—C7	179.46 (17)
C16—C7—C8—C9	73.4 (2)	C10—C11—C16—C7	55.4 (2)
C6—C7—C8—C9	−161.50 (18)	C12—C11—C16—C7	−68.2 (2)
O3—C8—C9—O5	−67.8 (2)	C8—C7—C16—C14	161.50 (19)
O4—C8—C9—O5	176.98 (16)	C6—C7—C16—C14	33.8 (3)
C7—C8—C9—O5	62.6 (2)	C8—C7—C16—C17	41.6 (2)
O3—C8—C9—C10	170.59 (18)	C6—C7—C16—C17	−86.1 (2)
O4—C8—C9—C10	55.4 (2)	C8—C7—C16—C11	−73.2 (2)
C7—C8—C9—C10	−59.0 (2)	C6—C7—C16—C11	159.07 (18)
O5—C9—C10—C20	95.7 (3)	C8—O4—C17—C16	1.0 (2)
C8—C9—C10—C20	−145.4 (2)	C14—C16—C17—O4	−152.40 (18)
O5—C9—C10—C11	−82.2 (2)	C11—C16—C17—O4	89.98 (19)
C8—C9—C10—C11	36.6 (3)	C7—C16—C17—O4	−28.1 (2)

### Hydrogen-bond geometry (Å, °)

**Table:**

*D*—H···*A*	*D*—H	H···*A*	*D*···*A*	*D*—H···*A*
O1W—H1W1···O4^i^	0.88	1.94	2.810 (2)	169
O2—H2···O2W^ii^	0.82	1.85	2.656 (3)	169
O1W—H2W1···O3^iii^	0.84	2.06	2.873 (3)	163
O3—H3···O2	0.82	1.71	2.525 (2)	171
O2W—H1W2···O8	0.95	2.03	2.950 (3)	164
O2W—H2W2···O10	0.85	1.91	2.760 (3)	179
O5—H5···O3^iv^	0.82	1.99	2.805 (2)	172
O6—H6···O9^v^	0.82	2.14	2.848 (2)	144
O7—H7···O1W^vi^	0.82	1.84	2.653 (3)	171
O10—H10···O7^vii^	0.82	2.29	3.011 (3)	147
O10—H10···O8^vii^	0.82	2.23	2.911 (3)	140
C1—H1A···O9	0.98	2.48	2.936 (3)	108
C1—H1A···O1^iii^	0.98	2.56	3.507 (3)	162
C7—H7A···O5	0.98	2.38	2.856 (3)	109
C12—H12A···O1^iii^	0.98	2.47	3.168 (3)	128
C17—H17A···O10^v^	0.97	2.60	3.428 (3)	144
C17—H17B···O6	0.97	2.50	2.929 (3)	107
C19—H19B···O2	0.96	2.56	2.957 (3)	105

Symmetry codes: (i) *x*+1, *y*+1, *z*; (ii) *x*−1/2, −*y*+1/2, −*z*+2; (iii) *x*+1/2, −*y*+1/2, −*z*+2; (iv) *x*+1/2, −*y*−1/2, −*z*+2; (v) −*x*+1, *y*−1/2, −*z*+3/2; (vi) *x*, *y*−1, *z*; (vii) −*x*+2, *y*+1/2, −*z*+3/2.
